# Standard protocol for the total red blood cell *Pig-a* assay used in the interlaboratory trial organized by the Mammalian Mutagenicity Study Group of the Japanese Environmental Mutagen Society

**DOI:** 10.1186/s41021-019-0121-z

**Published:** 2019-02-27

**Authors:** Satsuki Chikura, Takafumi Kimoto, Satoru Itoh, Hisakazu Sanada, Shigeharu Muto, Katsuyoshi Horibata

**Affiliations:** 1 Toxicology Research Department, Teijin Institute for Bio-medical Research, Teijin Pharma Limited, 4-3-2 Asahigaoka, Hino-shi, Tokyo, 191-8512 Japan; 2 Medicinal Safety Research Laboratories, Daiichi Sankyo Co., Ltd, 1-16-13, Kitakasai, Edogawa-ku, Tokyo, 134-8630 Japan; 3 Drug Research Center, Kaken Pharmaceutical Co., LTD, 301, Gensuke, Fujieda-shi, Shizuoka, 426-8646 Japan; 4 Safety Research Laboratories, Mitsubishi Tanabe Pharma Corporation, 2-2-50, Kawagishi, Toda-shi, Saitama, 335-8505 Japan; 5 Division of Genetics and Mutagenesis, National Institute of Health Sciences, 3-25-26 Tonomachi, Kawasaki-ku, Kawasaki, Kanagawa 210-9501 Japan

**Keywords:** *Pig-a* assay, Glycosylphosphatidylinositol, Flow cytometry, Red blood cells, In vivo gene mutation, CD59, HIS49

## Abstract

The *Pig-a* assay, a promising tool for evaluating in vivo genotoxicity, is based on flow cytometric enumeration of red blood cells (RBCs) that are deficient in glycosylphosphatidylinositol anchor protein. Various approaches for measuring *Pig-a* mutant cells have been developed, particularly focusing on measuring mutants in peripheral RBCs and reticulocytes (RETs). The *Pig-a* assay on concentrated RETs—the PIGRET assay—has the potential to detect genotoxicity in the early stages of a study. To verify the potential and usefulness of the PIGRET assay for short-term testing, we conducted an interlaboratory trial involving 16 laboratories organized by the Mammalian Mutagenicity Study Group of the Japanese Environmental Mutagen Society (MMS/JEMS). The collaborating laboratories assessed the mutagenicity of a total of 24 chemicals in rats using a single-treatment design and standard protocols for conducting the *Pig-a* assay on total RBCs (the RBC *Pig-a* assay) and the PIGRET assay. Here, we describe the standard protocol for the RBC *Pig-a* assay in detail.

## Background

The *Pig-a* assay is an in vivo gene mutation assay that uses the *Pig-a* gene as an endogenous reporter. The *Pig-a* assay has attracted attention as a potential mutation assay for regulatory safety assessments. In 2013, a workgroup of the International Workshop on Genotoxicity Testing (IWGT) reviewed data, protocols, and the state of assay validation, and published consensus statements on the current status and research needs for the assay [1]. Preparations are now underway for a new Organisation for Economic Cooperation and Development (OECD) test guideline for the in vivo *Pig-a* assay. In addition, the assay is recommended in the International Conference on Harmonization (ICH) guideline M7(R1), “Assessment and control of DNA reactive (mutagenic) impurities in pharmaceuticals to limit potential carcinogenic risk”, as a follow-up test for positive in vitro findings [2].

**Fig. 1:**
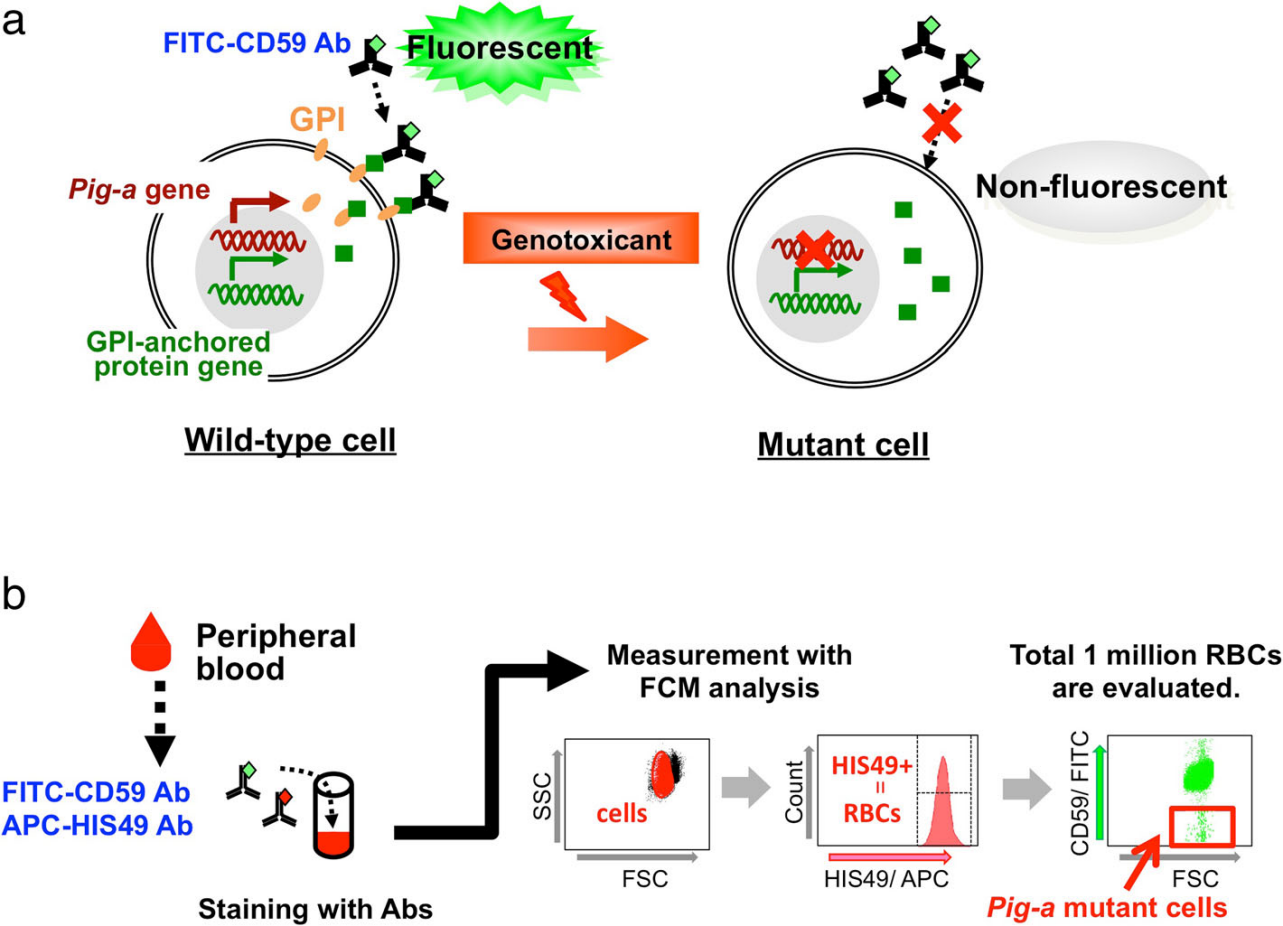
Principle of the *Pig-a* assay and flow cytometry analysis. **a** *Pig-a* is an essential gene for synthesis of the glycosylphosphatidylinositol (GPI) anchor. In wild-type cells, GPI anchors and CD59, a GPI-anchored protein marker, are synthesized independently and GPI tethers CD59 to the cell surface. However, in *Pig-a* mutant cells, CD59 proteins on the cell surface are reduced because GPI anchors are not synthesized due to *Pig-a* gene mutation(s). Thus, *Pig-a* mutant cells do not react with FITC-conjugated anti-CD59 antibodies while wild-type cells react to the antibodies and fluoresce. **b** Peripheral blood is stained with fluorescent-labeled antibodies. Cells are gated by light scatter and then analyzed by flow cytometry for HIS49 rat erythroid marker expression. HIS49-positive cells are further analyzed for CD59 expression and *Pig-a* mutant cells are detected as the FITC-negative population

*Pig-a* assays evaluate the mutagenic potential of chemicals by detecting phenotypic changes in cells caused by intracellular gene mutations. The *Pig-a* or phosphatidylinositol glycan class A gene (*Pig-a* in rodents, *PIG-A* in humans) codes for an enzyme essential for synthesis of the glycosylphosphatidylinositol (GPI) anchor [3–6]. GPI anchors tether many unique proteins, e.g., CD59, CD55, and CD48, to the surface of various cell types in humans and rodents [7, 8]. The *Pig-a* gene is located on the X chromosome in mammalian cells [3, 9] and is present as one functional copy per cell (the second copy is transcriptionally silenced in females). Thus, a single *Pig-a* gene mutation can result in a deficiency in GPI-anchored proteins at the cellular surface (Fig. 1a). Since the *Pig-a* assay uses an endogenous gene on the X chromosome for detecting mutations, transgenic rodents are not required. An additional advantage is that the *Pig-a* assay can often be integrated into existing genotoxicity and general toxicology studies as a combination assay.

The red blood cell (RBC) *Pig-a* assay can measure mutants that accumulate in whole peripheral blood as a result of repeat dosing [10]. Only a few microliters of peripheral blood from live animals are required to conduct the assay; thus, the mutagenicity risk of compounds may be evaluated longitudinally, in multiple samples collected from a single set of animals. When the *Pig-a* assay is conducted as part of long-term/chronic repeated dosing studies, that is, when aging animals are assayed, the RBC *Pig-a* assay might be preferable to other genotoxicity assays that require animal sacrifice (e.g., the transgenic rodent assay) or where genotoxicity responses do not accumulate (e.g., the Comet assay or the bone marrow micronucleus assay).

In vivo *Pig-a* assays were first described for rodents in 2008 [11–14]. Several methods using peripheral blood cells or bone marrow cells have been developed for mice and rats, but the rat peripheral blood method, particularly using RBCs, is most commonly used at present. Although there are multiple approaches for *Pig-a* assays, we used a method using the anti-rat erythroid marker HIS49 in this protocol. The RBC *Pig-a* assay was conducted using anti-HIS49 to identify erythrocytes and a flow cytometer (FCM) to evaluate mutant frequency (MF) in erythrocytes from peripheral blood. The *Pig-a* gene is essential for GPI synthesis; thus, mutants are identified as HIS49-positive cells with reduced GPI-anchored protein (CD59 in this assay) on the cell surface (Fig. 1a). Comparing with the others, this is the simplest protocol for *Pig-a* assay, that is, this requires less blood sample volumes and less experimental procedures. The technical hurdle for FCM setup is also lower, so this protocol is suitable for first time users of *Pig-a* assay.

The RBC *Pig-a* assay has been used in two collaborative studies conducted by Japanese research groups. The first of these studies was conducted in a trial supported by the Japan Health Sciences Foundation (JHSF). The collaborating laboratories defined standardized gating rules for CD59-negative cells and examined the interlaboratory transferability and reproducibility of the RBC *Pig-a* assay [15–20]. The second collaborative study was organized by the Mammalian Mutagenicity Study Group of the Japanese Environmental Mutagen Society (MMS/JEMS). The collaborators confirmed that significant increases in *Pig-a* MF were observed after a single administration of typical mutagens. In addition, the results of the MMS/JEMS collaborative study demonstrated the excellent reproducibility and high transferability of the assay [21].

In the present paper, we describe the procedure for the rat RBC *Pig-a* assay that was validated in the MMS/JEMS collaborative study. An outline of the assay is shown in Fig. 1b. Peripheral blood is collected from rats and mixed with an anticoagulant. The blood samples are stained with fluorescent-labeled anti-CD59 antibody and fluorescent-labeled antibody to an erythroid marker (HIS49) and, then, analyzed using an FCM. At least one million RBCs are analyzed for each sample, and the frequency of CD59-negative cells, which is taken to be the *Pig-a* MF, is calculated.

## RBC *Pig-a* assay standard protocol

### Instruments

The standard procedure for the RBC *Pig-a* assay uses an FCM equipped with blue and red lasers and its corresponding analysis software. Here, we describe a procedure with a FACSCantoII FCM (BD Biosciences) equipped with 488 nm blue and 633 nm red lasers and FACSDiva software (BD Biosciences) as an example.

A single-laser FCM can be used if an alternative fluorescent label (e.g., PerCP-Cy5.5-conjugated) is used on the anti-rat erythroid antibody. See Notes section (a) and the article by Kikuzuki et al. [22].

### Chemicals and materials

FITC-conjugated anti-rat CD59 antibody (FITC-CD59 Ab, clone TH9, BD Biosciences) and APC-conjugated anti-rat erythroid marker antibody (APC-HIS49 Ab, clone HIS49, BD Biosciences) are obtained commercially. Phosphate buffered saline (Ca- and Mg-free, PBS) is required to dilute blood samples and antibodies.

EDTA-2 K solution (12 mg/mL) is used as an anti-clotting reagent for tail vein blood collection.

### Animals and dosing

Both male and female rats can be used for this assay [23–25]. Groups of six were recommended by the 6th IWGT *Pig-a* Workgroup [1]. The maximum dose and lower doses should be selected according to the criteria laid out in the OECD Test Guidelines for in vivo genotoxicity and general toxicity studies (e.g., TG407 [26], TG474 [27], and TG488 [28]).

The positive control group is not required to be the same size as the test group; however, it is necessary to judge whether the assay is performed properly (see Notes section (b)). *N*-Nitroso-*N*-ethylurea (Cas# 759-73-9, ENU) may be used as a positive control compound; a single dose of 40 mg/kg ENU induces significant increases in *Pig-a* MF. Dissolve ENU in warm PBS (pH adjusted to 6.0–6.1, 37 °C) and filter the solution to remove undissolved particles. Keep away from light and use within 2 h.

### Blood collection and preservation

#### Blood collection

Blood is collected from rats prior to and at appropriate times after the administration of the test compound. In the MMS/JEMS collaborative study, the RBC *Pig-a* assays were conducted prior to and 1, 2, and 4 weeks after a single administration.

About 10 μL peripheral blood are collected from a tail vein and mixed well with 12 mg/mL EDTA-2 K solution at a ratio of 9:1 or 10:1. To prepare control samples for gate adjustments, an additional 10 μL blood should be collected from one of the animals in the negative or vehicle control groups. It is also possible to collect blood from the abdominal aorta with vacutainer blood collection tubes containing anti-coagulants (e.g., EDTA or heparin). Store blood samples on ice or in a refrigerator (2–8 °C).

Coagulation of the blood samples has a negative impact on *Pig-a* assay data, and may cause a false positive result due to poor staining. The blood sampling method is left to the operator’s discretion as long as blood coagulation is avoided.

#### Storage of blood samples

Refrigerated blood samples should be used for the *Pig-a* assay within seven days of collection. Prior to storage, the tubes are centrifuged briefly to collect all the blood, including blood adhering to the lid and the wall of the tubes, into a single volume at the bottom and prevent the blood from drying out. Then, the tubes are tightly capped and stored in a dark refrigerator (2–8 °C).

### Blood processing for the RBC *Pig-a* assay

#### Preparation of master mix

One μg FITC-CD59 Ab and 0.133 μg APC-HIS49 Ab solution for each 3 μL blood sample (plus some extras) were combined in a master mix solution. Specifically, 2 μL FITC-CD59 Ab stock solution (0.5 mg/mL) and 2 μL diluted APC-HIS49 Ab solution (0.2 mg/mL stock solution is diluted three-fold with PBS to 0.0667 mg/mL) are mixed per sample. Four μL of the master mix solution are used to stain each sample.

#### Staining assay samples

Dispense 0.2 mL PBS into each FCM sample tube. Ensure that the PBS is at the bottom of the sample tube, taking care to prevent PBS from adhering to the wall of the tube. Since blood might separate during storage, mix the blood samples well by tapping, pipetting, or vortexing before dispensing. Take 3 μL blood using a micropipette and completely expel it into the PBS in the sample tube, taking care to prevent blood from adhering to the wall of the sample tube. Rinse blood in the pipette tip by pipetting and vortex the sample tube for several seconds.

Add 4 μL of the master mix prepared above to each assay sample, taking care to prevent the antibody from adhering to the wall of the sample tube. Rinse the antibody in the tip by pipetting. After adding the master mix solution to all samples, vortex the sample tubes for several seconds. If blood splashes on the upper part of the tube, mix well until it is mixed with antibodies completely, or transfer the sample solution to a new tube.

Incubate samples for 1 h at room temperature in the dark. After incubation, mix the samples again by vortexing and, then, centrifuge at approximately 1700×g for 5 min at room temperature. Remove the supernatant with an aspirator or a pipette while tilting the tubes.

Loosen the blood cell pellet by gently tapping the bottom of the tube. It is critical to thoroughly disperse the pellet at this point to keep cells from clumping. Add 0.5–1.0 mL PBS, resuspend the blood cells and vortex for several seconds. Adjust the volume of PBS to attain the desired sample flow rate on the FCM; see the ‘Data collection’ section below. Keep the samples at room temperature in the dark until FCM analysis.

#### Staining control samples used for gate adjustment

A non-stained sample, a CD59 single-stain sample, and a HIS49 antibody single-stain sample are prepared. Dispense 0.2 mL PBS into sample tubes and take 3 μL of blood collected from an animal in the negative or vehicle control group. Expel it completely into the PBS in each sample tube.

Add 2 μL FITC-CD59 Ab stock solution to the CD59 single-stain sample tube. Add 2 μL diluted APC-HIS49 Ab to the HIS49 single-stain sample tube. The CD59 single-stain sample and the HIS49 single-stain sample contain 1 μg and 0.133 μg of each antibody, respectively. Incubate the two single-stain samples and the non-stained sample for 1 h, centrifuge at approximately 1700×g for 5 min at room temperature and, then, resuspend as described above for assay samples.

### Flow cytometry

The following steps vary depending on the FCM model and analysis software; the procedures described below describe using a BD FACSCantoII FCM and BD FACSDiva software, but are relevant for most models. The details of each procedure and gate settings can be adjusted at each facility to fulfill the acceptance criteria for the analysis.

#### Cytometer startup and plot creation

Start up the FCM as described in the instruction manual. Perform quality control to ensure that the instrument is in optimal condition. Keep the flow cell and fluid lines clean to prevent detecting nonspecific events.

**Fig. 2:**
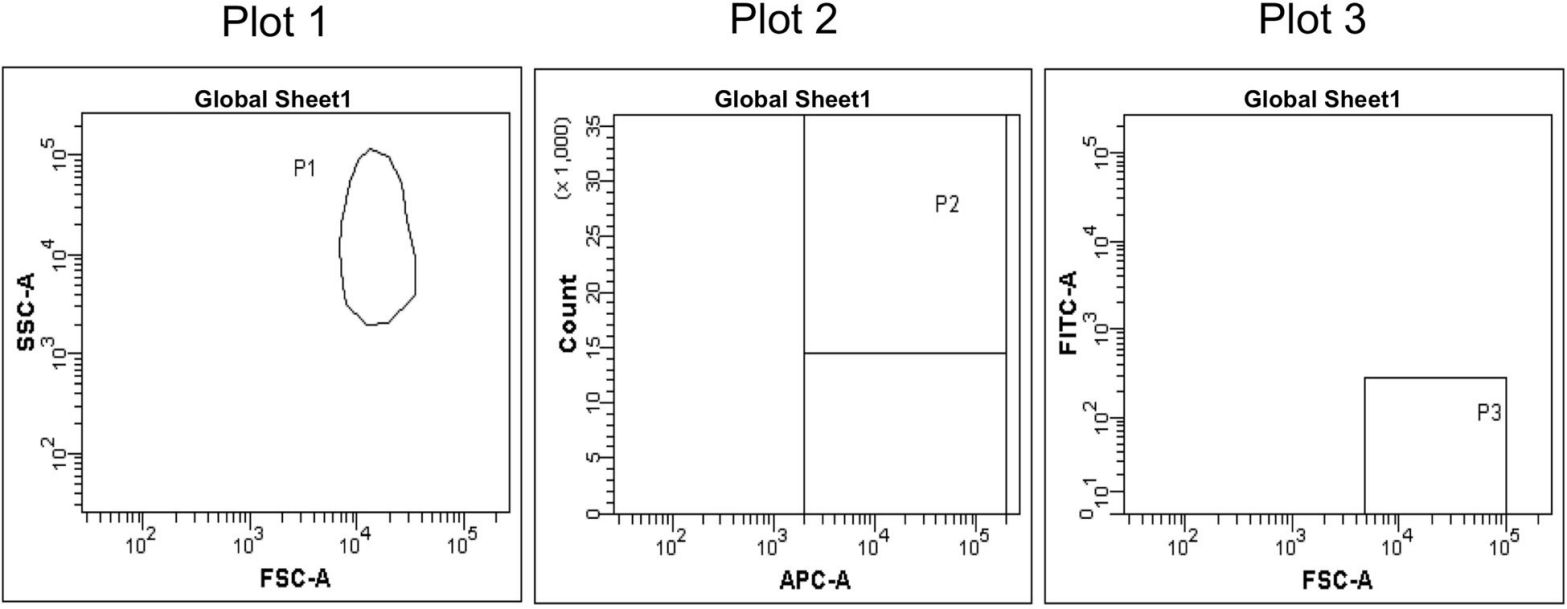
Plots for the RBC *Pig-a* assay. Create dot plots of FSC vs SSC (Plot 1) and FSC vs FITC (Plot 3) and a histogram of APC fluorescence (Plot 2). Set FSC and SSC in the log scale. When using digital flow cytometry systems, set area scaling factors to appropriate values and use the area measurement

Create a worksheet and three plots, as shown in Fig. 2: dot plots of forward scatter (FSC) vs side scatter (SSC) and FSC vs FITC, and a histogram for APC fluorescence. Set FSC and SSC in the log scale. When using analysis software such as FACSDiva, set the area scaling factor at an appropriate value so that the results of the analyses using area will be accurate. To evaluate only a single-cell population and eliminate nonspecific data, an FSC-H/FSC-W dot plot can be included in addition to the basic plots defined in Fig. 2. Analog instruments such as the FACSCalibur FCM do not have this function; therefore, an area scaling factor and the FSC-H/FSC-W plot are not used.

#### Creating gates with the non-stained sample

**Fig. 3:**
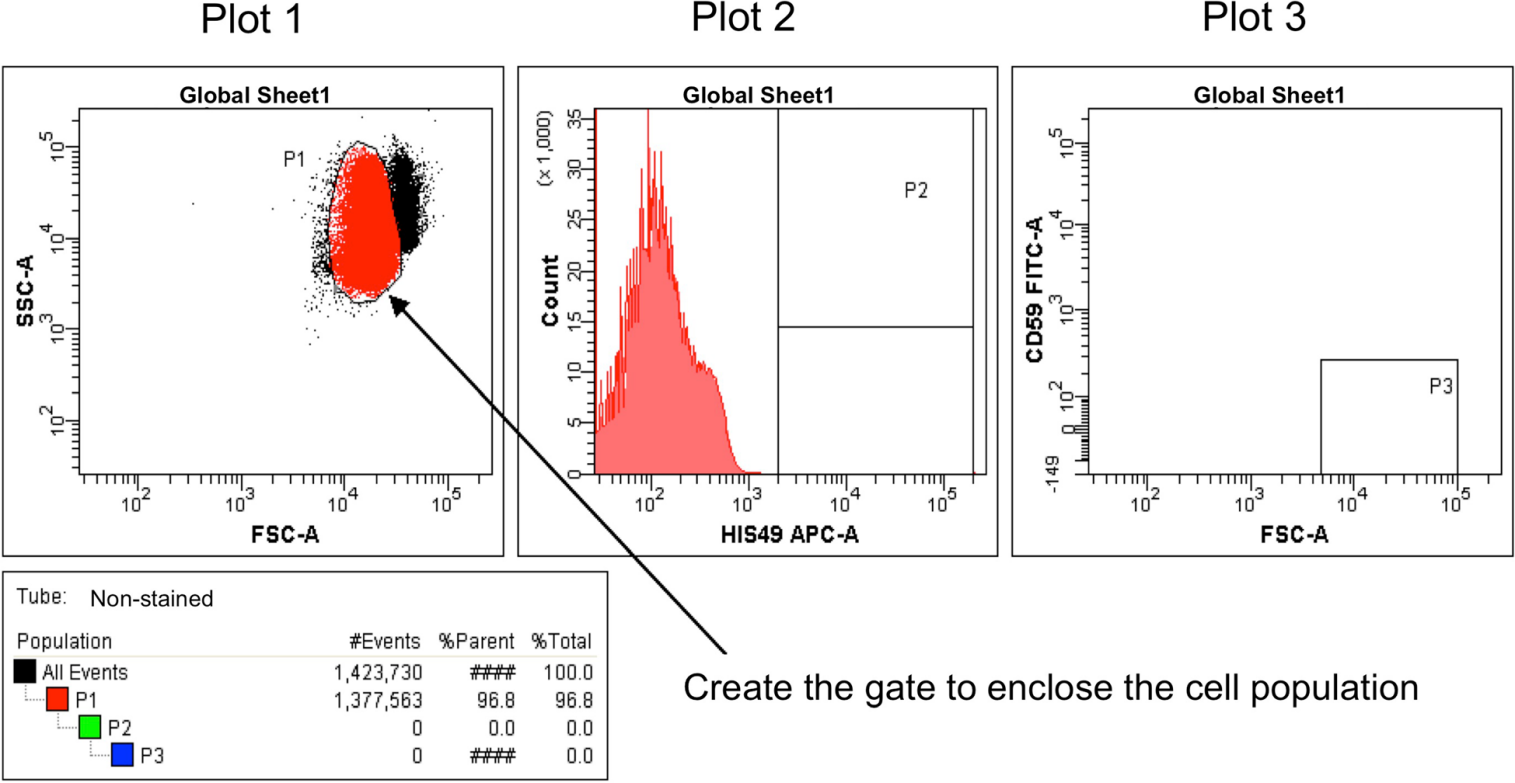
Creating gates and adjustments with the non-stained control sample. Create P1, P2, and P3 gates and confirm that the P2 and P3 gates are subpopulations of the P1 and P2 gates, respectively. Display only the parent’s population on the subsequent plot (e.g., only the P1 population is displayed in Plot 2)

Place the non-stained sample in the FCM and start acquiring and previewing data (without recording). During data acquisition, adjust the photomultiplier tube (PMT) voltage so that the cell population is plotted in the upper right quadrant of the FSC/SSC plot, as shown in Plot 1 of Fig. 3. Create a P1 gate by enclosing the cell population in Plot 1 using a polygon or a freeform gate tool. If applicable, a threshold option can be set on FCS and SSC.

Then, create a P2 gate in Plot 2 using an interval gate tool, as shown in Plot 2 of Fig. 3. Display only the P1 gate population on Plot 2 and create the P2 gate as a subset of the P1 gate. Confirm that the new population (P2 gate) appears indented below the selected population (P1 gate) in the Population Hierarchy view. Create a P3 gate in Plot 3 using a rectangle gate tool, as shown in Plot 3 of Fig. 3. Display only the P2 gate population in Plot 3 and create the P3 gate as a subset of the P2 gate. At this point, few cells are detected in Plot 3 since the cells are not stained with antibodies. Confirm that the P3 gate appears indented below the P2 gate in the Population Hierarchy view.

#### Adjusting gates with single-stain samples

While loading each single-stain sample, adjust the PMT voltages of the APC and FITC signals so that the positive or negative cell population shifts to an appropriate position in the P2 and P3 plots.

**Fig. 4:**
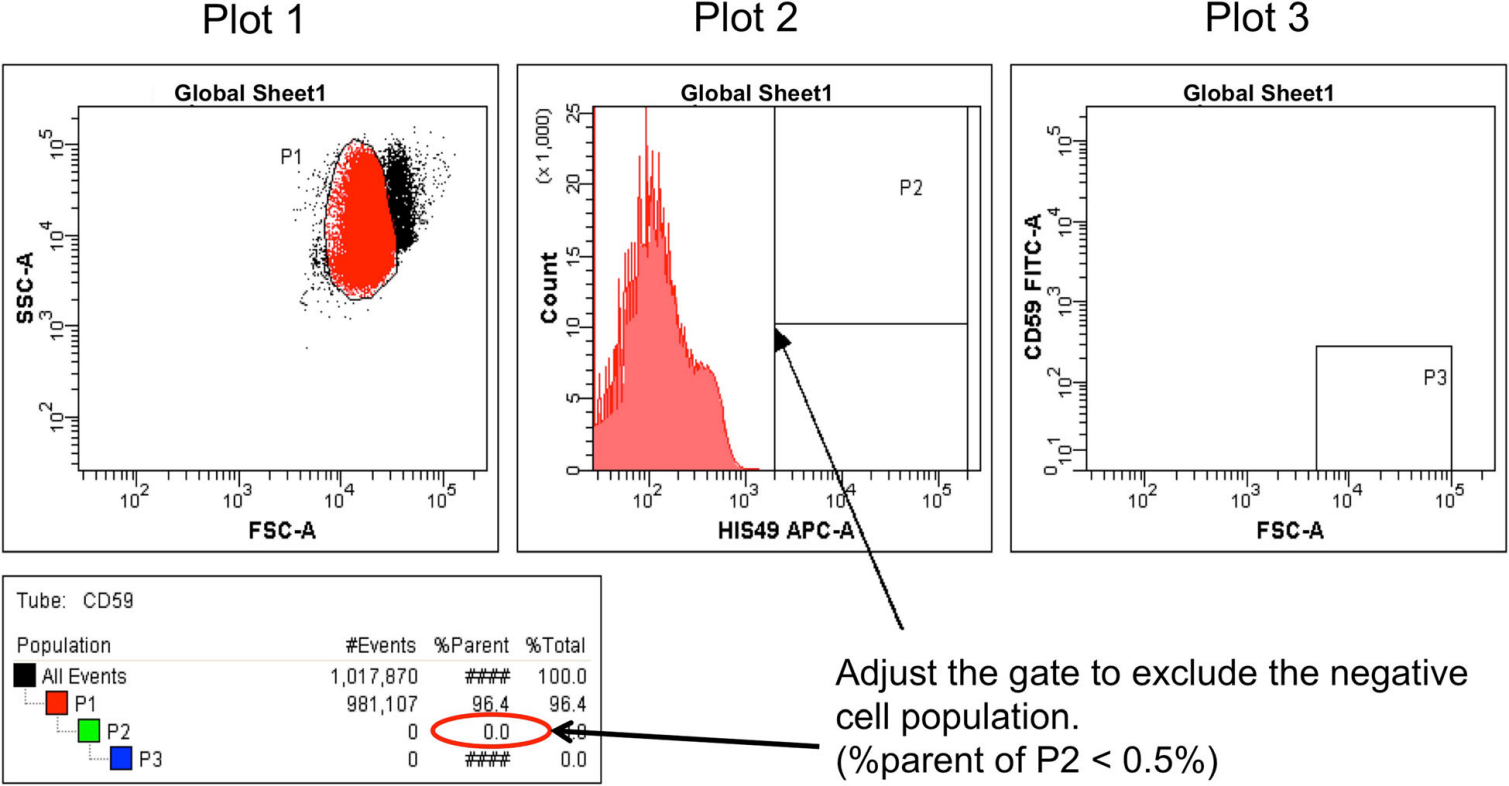
Gate adjustment with the CD59 single-stain sample. Adjust the P2 gate to exclude the negative cell population; insure that %parent of P2 is below 0.5%

Adjust the position of the P2 gate using the CD59 single-stain sample. Move the P2 gate to include less than 0.5% of the APC-negative population (Fig. 4).

**Fig. 5:**
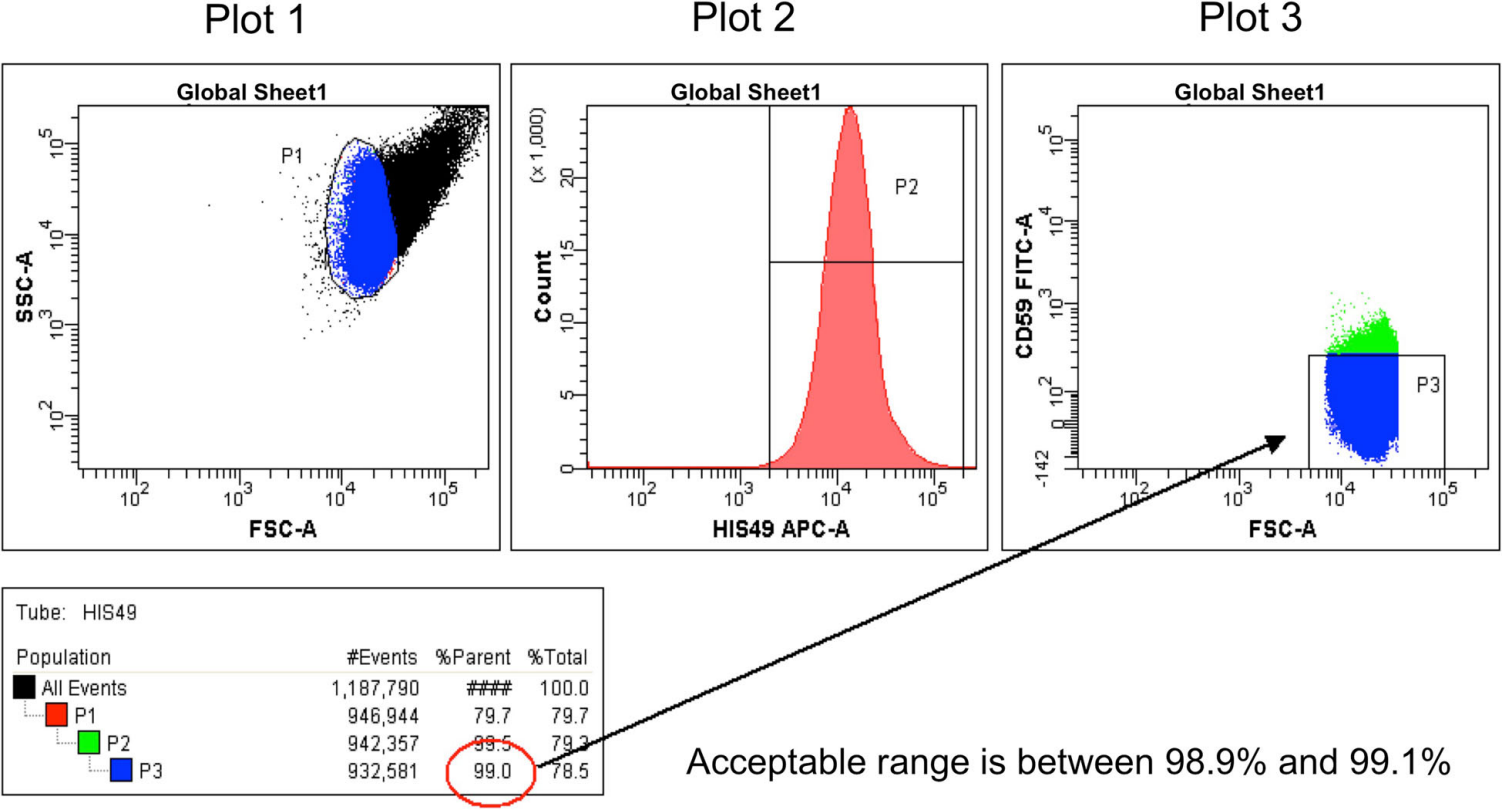
Gate adjustment with the HIS49 single-stain sample. Adjust the height of the P3 gate to include 99.0 ± 0.1% of the CD59-negative cell population. Make sure that the P3 gate reaches the x-axis. If applicable, select “Bi-exponential” for the y-axis (FITC intensity scaling) in the Plot Inspector

Next, optimize the P3 gate following the rule set in our previous collaborative study, the JHSF study [15]. Gate P3 is critical for the accurate detection of CD59-negative cells. At this point, use the HIS49 single-stain sample which is detected in the P3 region as a CD59-negative cell population. Adjust the P3 gate to include 99.0% (acceptable range is between 98.9 and 99.1%) of the CD59-negative cell population in Plot 3 (Fig. 5). Confirm that the P3 gate reaches the x-axis. If applicable, select “Bi-exponential” for the y-axis (FITC intensity scaling) in the Plot Inspector.

Finally, acquire and record data for control samples in the following order: HIS49 single-stain sample, non-stained sample, and CD59 single-stain sample. Count at least one million cells in the P1 region for the non-stained and the CD59 single-stain samples and in the P2 region for the HIS49 single-stain sample. If the single-cell population is defined, count cells in the single-cell region instead of the P1 region.

Once the PMT voltages and gates have been set appropriately and saved as a protocol, subsequent analyses can be performed. Before acquiring data with the saved protocol, fine-tune the PMT voltages and the gate settings. Make sure that the rule for the CD59-negative cell region has been strictly applied to the P3 gate setting; that is, that the P3 gate is set to include 99.0 ± 0.1% of the CD59-negative cell population using the HIS49 single-stain sample (see Notes section (c)).

#### Data collection

Immediately before placing an assay sample on the FCM, thoroughly mix the sample by vortexing. If cell clumps are present in the sample tubes, filter the sample solutions with a cell strainer. It is advisable to collect and save all events to the database rather than certain cell populations (e.g., cells in the P1 region), so that the complete data are available for re-analysis if needed.

Maintain the sample flow rate at 10,000 events/sec or below by adjusting the event rate on the instrument or the cell concentration of each sample. By adjusting to an appropriate flow rate, nonspecific data are reduced and highly efficient sample analysis is achieved. Acquire and record data for at least one million cells in the P2 region (HIS49-positive cells: total RBCs).

If a large number of cells are detected in the P3 region (CD59-negative cells), load PBS or purified water to wash the fluid lines and prevent contaminating the cells in the next sample. After washing, acquire data from the next sample, confirm that the events fall within the appropriate gates and, then, continue to record data.

### Data analysis

#### Calculation of *Pig-a* MF

*Pig-a* MF is calculated according to the following formula:

$$ Pig-a\ \mathrm{MF}\ \left(\times {10}^{-6}\right)=\frac{\mathrm{Number}\ \mathrm{of}\ \mathrm{CD}59\ \mathrm{negative}\ \mathrm{RBCs}\ \left(\mathrm{cells}\ \mathrm{in}\ \mathrm{the}\ \mathrm{P}3\ \mathrm{region}\right)}{\mathrm{Number}\ \mathrm{of}\ \mathrm{total}\ \mathrm{RBCs}\ \left(\mathrm{cells}\ \mathrm{in}\ \mathrm{the}\ \mathrm{P}2\ \mathrm{region}\right)}\times {10}^6 $$

#### Acceptance criteria for the assay

The negative/vehicle and positive control groups are required to satisfy the criteria below (see Notes section (b)):

- The average *Pig-a* MF in the negative/vehicle control group is 10 × 10^−6^ or below.
- The *Pig-a* MF of each animal in the positive control group is significantly higher than that in the negative control group (10 × 10^−6^ or above is adequate).

### Statistical analysis

Statistical analyses of *Pig-a* MF data are performed following the method described in the IWGT report [1].

An offset of 0.1 is added to each *Pig-a* MF value (expressed as mutants × 10^−6^ total RBCs) because *Pig-a* MF values of zero are occasionally observed, and the values are log (10) transformed. Then, transformed *Pig-a* MF values are analyzed by Bartlett’s test for homogeneity of variance among the groups. If the group variance is determined to be homogeneous, the significance of increases in treated rats relative to negative control groups is analyzed using Dunnett’s multiple comparison test. If Bartlett’s test indicates heterogeneous variance, the nonparametric Dunnett’s multiple comparison test (Steel test) is used. Significance is evaluated at the 5% level using a one-tailed test for increases relative to the negative or vehicle control.

### Tips for conducting the RBC *Pig-a* assay

The RBC *Pig-a* assay has just two simple steps to obtain data; the first step is mixing each blood sample and antibodies and the second step is analyzing the samples using an FCM. The method is simple; however, it is important to stain the blood samples carefully and set up the FCM correctly. Any unstained blood in the sample tube might cause a false positive result in the *Pig-a* assay because unstained cells are recognized as CD59-negative (mutant) cells. The rule for CD59 negative gate-setting, which is defined as containing 99.0 ± 0.1% cells using the HIS49 single-stained sample, is effective for reducing variability in the measurement of CD59-negative cells (*Pig-a* MF) [15]. To evaluate compounds correctly, insure that the PMT voltages are adjusted appropriately and that the CD59-positive (wild-type) population has sufficient FITC intensity. If normal (wild-type) cells have insufficient fluorescence intensity in spite of the PMT voltage adjustment, it is better to change the antibody. Keep the flow cell and fluid lines clean to prevent the detection of nonspecific particles.

**Fig. 6:**
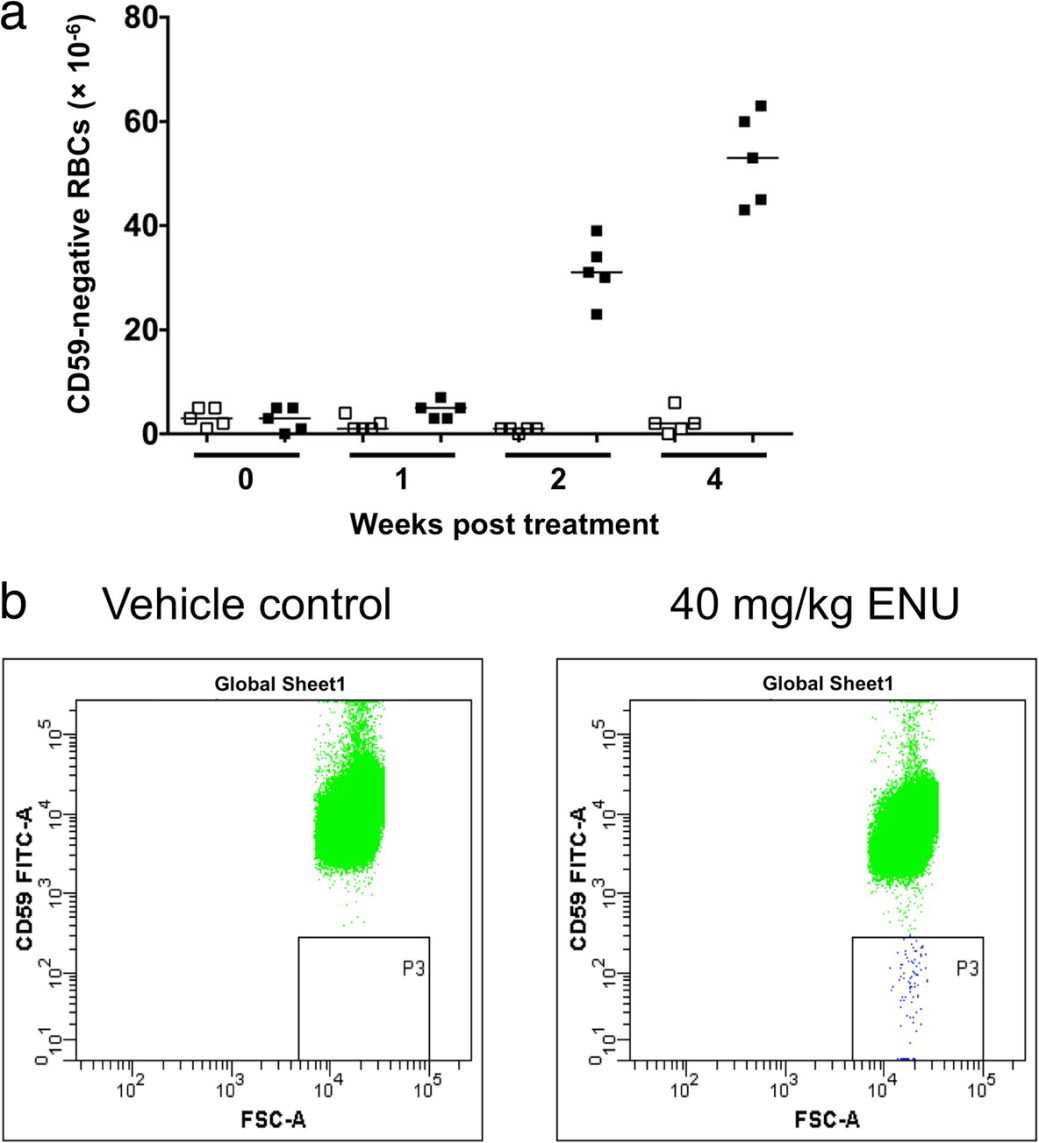
Example RBC *Pig-a* assay results and typical flow cytometer plots. **a** RBC *Pig-a* assays were conducted pre-treatment (0), and at 1, 2, and 4 weeks after a single administration of PBS (vehicle control, open symbols) or 40 mg/kg ENU (closed symbols). This figure was reproduced from Genes and Environment 36, 199–202, 2014 [20] and modified. **b** Typical plots measuring *Pig-a* mutant cells (CD59-negative cells) from vehicle control and ENU treatment groups

When each facility establishes and validates RBC *Pig-a* assay techniques on-site, it is desirable to conduct an assay on rats treated with a single dose of 40 mg/kg ENU and confirm that the *Pig-a* MF increases significantly at 2 or 4 weeks after administration, as shown Fig. 6. The average *Pig-a* MF in the negative control group should be consistently 10 × 10^− 6^ or below.

### Notes

- When using a single-laser FCM, use PerCP-Cy5.5-labeled erythroid marker, which can be used with FITC- and PE-labeled reagents for analyses using a single-laser FCM. Kikuzuki et al. conducted the *Pig-a* assay with an Epics XL equipped with SYSTEM II software in the MMS/JEMS collaborative study [22], and their results satisfied the acceptance criteria. For a single-laser FCM, alter the procedures as follows.
  PerCP-Cy5.5-conjugated rat erythroid marker (clone HIS49) is prepared. Add 0.8 μg PerCP-Cy5.5-conjugated HIS49 antibody per sample; if PerCP-Cy5.5-conjugated HIS49 antibody stock solution is 0.2 mg/mL, the master mix is prepared by mixing 2 μL FITC-CD59 Ab stock solution and 4 μL PerCP-Cy5.5-conjugated HIS49 antibody per sample for the total number of assay samples. Then, add 6 μL master mix to each sample tube. Create a histogram of PerCP-Cy5.5 fluorescence instead of APC and adjust the gates in the same manner as described in the ‘Flow cytometry’ section. If needed, calculate and apply compensation values.
- Acceptance criteria in this protocol are intended for first time users for *Pig-a* assays.
  Positive control: A positive control group is required as acceptance criteria to validate the *Pig-a* assay technique on-site. According to the IWGT recommendations [1], a positive control group is not considered mandatory in case that an appropriate standard that “mimics” mutants is used each time FCM analysis.
  Negative/Vehicle control: After validating the *Pig-a* assay technique and gathering enough historical data on-site, the acceptance criteria can include comparison of the negative control values with the historical negative control distribution for the laboratory.
- If a FACSCalibur FCM and FlowJo software are used, the instrument settings for the analysis can also be confirmed based on the peak Ch value. By setting the peak Ch value for the cell population that is positive for HIS49 (for example, a fixed value of 400–430) in advance, the instrument settings can be adjusted by simply changing the PMT voltage without redefining the gate when there is any deviation from the previous analysis. When using FACSDiva, a similar setting can be developed by the mean value in the statistics view.

## Conclusions

The *Pig-a* assay is an attractive in vivo gene mutation assay, using an endogenous reporter gene, that has shown potential for conducting regulatory safety assessments. The RBC *Pig-a* assay requires only a few microliters of blood from animals, a property that facilitates developing longitudinal data from single groups of animals and integrating the assay with other genotoxicity and general toxicity tests. The protocol described here was validated by the MMS/JEMS collaborative study and the results were reported in a special issue of Mutation Research (Vol. 811, 2016). We expect that studies using this protocol will provide important information for further development of the *Pig-a* assay.

## Abbreviations

APC-HIS49 Ab: APC-conjugated anti-rat erythroid marker antibody
ENU: *N*-Nitroso-*N*-ethylurea
FCM: Flow cytometer
FITC-CD59 Ab: FITC-conjugated anti-rat CD59 antibody
FSC: Forward scatter
GPI: Glycosylphosphatidylinositol
IWGT: International Workshop on Genotoxicity Testing
JHSF: Japan Health Sciences Foundation
MF: Mutant frequency
OECD: Organisation for Economic Cooperation and Development
PBS: Phosphate buffered saline
PMT: Photomultiplier tube
RBC: Red blood cell
RET: Reticulocyte
SSC: Side scatter
